# Anatomical Correlates of Uncontrollable Laughter With Unilateral Subthalamic Deep Brain Stimulation in Parkinson’s Disease

**DOI:** 10.3389/fneur.2018.00341

**Published:** 2018-05-25

**Authors:** Yonglu Huang, Joshua P. Aronson, Julie G. Pilitsis, Lucy Gee, Jennifer Durphy, Eric Steven Molho, Adolfo Ramirez-Zamora

**Affiliations:** ^1^Department of Neurology, Dartmouth-Hitchcock Medical Center, Lebanon, NH, United States; ^2^Department of Neurology, The Second Hospital of Anhui Medical University, Hefei, China; ^3^Department of Surgery, Section of Neurosurgery, Dartmouth-Hitchcock Medical Center, Lebanon, NH, United States; ^4^Department of Neurosurgery, Albany Medical Center, Albany, NY, United States; ^5^Department of Neurology, Albany Medical Center, Albany, NY, United States; ^6^Department of Neurology, Center for Movement Disorders and Neurorestoration, University of Florida, Gainesville, FL, United States

**Keywords:** subthalamic nucleus, substantia nigra reticulate, uncontrolled laughter, deep brain stimulation, electrode, Parkinson’s disease

## Abstract

**Introduction:**

Subthalamic nucleus deep brain stimulation (STN-DBS) is a well-established treatment for the management of motor complications in Parkinson’s disease. Uncontrollable laughter has been reported as a rare side effect of STN stimulation. The precise mechanism responsible for this unique phenomenon remains unclear. We examined in detail the DBS electrode position and stimulation parameters in two patients with uncontrollable laughter during programming after STN-DBS surgery and illustrated the anatomical correlates of the acute mood changes with STN stimulation.

**Case report:**

Unilateral STN-DBS induced uncontrollable laughter with activation of the most ventral contacts in both patients. However, the location of the electrodes responsible for this adverse effect differed between the patients. In the first patient, the DBS lead was placed more inferiorly and medially within the STN. In the second patient, the DBS lead was implanted more anteriorly and inferiorly than initially planned at the level of the substantia nigra reticulata (SNr).

**Conclusion:**

Unilateral STN-DBS can induce acute uncontrollable laughter with activation of electrodes located more anterior, medial, and inferior in relationship with the standard stereotactic STN target. We suggest that simulation of ventral and medial STN, surrounding limbic structures or the SNr, is the most plausible anatomical substrate responsible for this acute mood and behavioral change. Our findings provide insight into the complex functional neuroanatomical relationship of the STN and adjacent structures important for mood and behavior. DBS programming with more dorsal and lateral contacts within the STN should be entertained to minimize the emotional side effects.

## Introduction

Subthalamic nucleus deep brain stimulation (STN-DBS) is an established and effective procedure for the treatment of motor complications in Parkinson’s disease (PD). Because of its small size and functional organization, STN-DBS may cause a variety of sensory and emotional changes associated with stimulation of limbic regions and other surrounding structures (1–3). While there are numerous reports of neuropsychiatric and affective changes associated with STN-DBS, it has been difficult to delineate the specific functional neuroanatomy in cases of acute enlightened mood (4, 5). In addition, reports lack detailed information of active contacts or clear post-operative imagining allowing assessment of potential factors and structures responsible for this uncommon side effect (4, 5). Marked differences among patients, methods, and inconsistent reports limit concrete conclusions (Table 1).

**Table 1:** Post-operative STN-DBS contact.

STN-DBS contact number
**Patient 1 right STN-DBS (X, Y, Z)**
Contact 0: 9.48 mm lateral to MC, 4.32 mm posterior to MC; 5.43 mm inferior to MC
Contact 1: 9.85 mm lateral to MC, 3.06 mm posterior to MC; 2.81 mm inferior to MC
Contact 2: 10.21 mm lateral to MC, 2.30 mm posterior to MC; 1.95 mm inferior to MC
Contact 3: 10.66 mm lateral to MC, 1.34 mm posterior to MC; 0.70 mm inferior to MC

**Patient 2 left STN-DBS (X, Y, Z)**
Contact 0: 11.64 mm lateral to MC, 1.61 mm posterior to MC; 6.10 mm inferior to MC
Contact 1: 12.24 mm lateral to MC, 0.21 mm posterior to MC; 4.38 mm inferior to MC
Contact 2: 12.62 mm lateral to MC, 1.02 mm anterior to MC; 2.61 mm inferior to MC
Contact 3: 13.13 mm lateral to MC, 2.03 mm anterior to MC; 1.20 mm inferior to MC

*X, lateral coordinate; Y, A-P coordinate; Z, vertical coordinate*.

DBS provides a unique opportunity to analyze the effects of electrical stimulation in neuronal structures and its connections. Stimulation of the ventral–medial STN region might result in spread of current into the limbic STN territories and therefore contribute to acute mood and behavioral changes (3, 4, 6). In this report, we presented two patients with reproducible uncontrollable, “mirthful” laughter after STN-DBS and reviewed prior reports of similar symptoms reported in the literature (4, 5, 7). Uncontrollable laughter has been considered as a pseudobulbar effect or a neuropsychiatric effect of STN-DBS surgery (8–10). We aim to assess the location of responsible contact to provide further insight into the complex basal ganglia mechanisms driving emotional responses in PD.

## Case Presentation

### Case 1

A 52-year-old right-handed man with a history of idiopathic PD for 20 years presented with severe motor fluctuations including end-of-dose wearing off, freezing of gait, and peak-dose dyskinesia. He had a history of impulse-control disorders associated with dopamine agonists and a history of depression. After comprehensive multidisciplinary DBS assessment, he had undergone staged bilateral STN-DBS based on the ability to reduce dopaminergic medications with the target compared with the GPi. He underwent unilateral left STN-DBS placement (Activa SC DBS implanted with a 3389 Medtronic lead) followed by right STN-DBS 6 months later. Microelectrode recording was conducted sequentially, and typical STN-neuronal discharges were recorded. Kinesthetic cells were encountered and macrostimulation after final placement of the lead was unremarkable with non-specific side effects including dizziness at high levels followed by corticobulbar side effects with dysarthria. During initial DBS monopolar review 1 month after surgery, he noticed a sudden onset of “giddiness and euphoria” best described as a need to laugh that was overwhelming and precluded him from talking when activating right STN contact 0. The programming was done unilaterally and the contralateral DBS was off during the session. Symptoms appeared around 3.0 V [pulse width (PW) of 60 µs and frequency of 140 Hz] and became increasingly prominent as voltage was increased to 3.5 V. The patient reported a feeling of happiness and joy associated with involuntary laughing. Despite the jovial nature of his symptoms, he was uncomfortable and the sensation was not pleasant. He reported mild nausea and recurrence of euphoric, loose, and giddy feelings when activating adjacent contact 1 at 3.8 V. At 4.3 V, this feeling became persistent and more intense. Partial benefit in Parkinsonism was observed with ventral contacts and the patient developed right foot dyskinesia, mild reduction in bradykinesia, and reduction in rigidity around 3.5 V. The rest of his monopolar review was unremarkable with improvement in Parkinsonism with contacts 2 and 3 and dyskinesia at 2.5 V with contact 2. The uncontrollable laughter was reproducible after 6 months. Corticospinal side effects and non-specific dizziness were the most common side effects associated with stimulation of contacts 2 and 3. The final programming settings were bipolar with contact 3 positive, contact 2 negative, PW 90 µs, and frequency 140 Hz. His UPDRS-III score off medications at 1 year improved from 42 to 15 points.

### Case 2

Patient was a 39-year-old right-handed woman who received bilateral STN stimulation for progressive Parkinsonism. She reported progressing symptoms started 6 years ago with development of early motor fluctuations and dyskinesia. She underwent simultaneous placement of bilateral STN-DBS 5 years after her diagnosis of PD (Activa SC DBS, implanted with a 3389 Medtronic lead). While performing monopolar review 1 month post-operatively, she noted sudden and uncontrollable laughter when activating contact 0 at 2.0 V (PW of 60 µs and frequency of 140 Hz) on the left side. Her symptoms were intermittent, with sudden laughter at higher voltages and episodes of normal mood. Similarly, contralateral DBS was off during the programming session. Uncontrollable laughter was reproducible with unilateral left STN stimulation but did not occur with right STN stimulation. Programming other contacts provided improvement in parkinsonism without acute mood effects. The final programming settings were bipolar with contact 3 positive, contact 2 negative, amplitude 2.5 V, PW 60 µs, and frequency 140 Hz. Her UPDRS-III score off medications at 1 year improved from 51 to 26 points.

### Anatomical Location of the Leads

**Figure 1:**
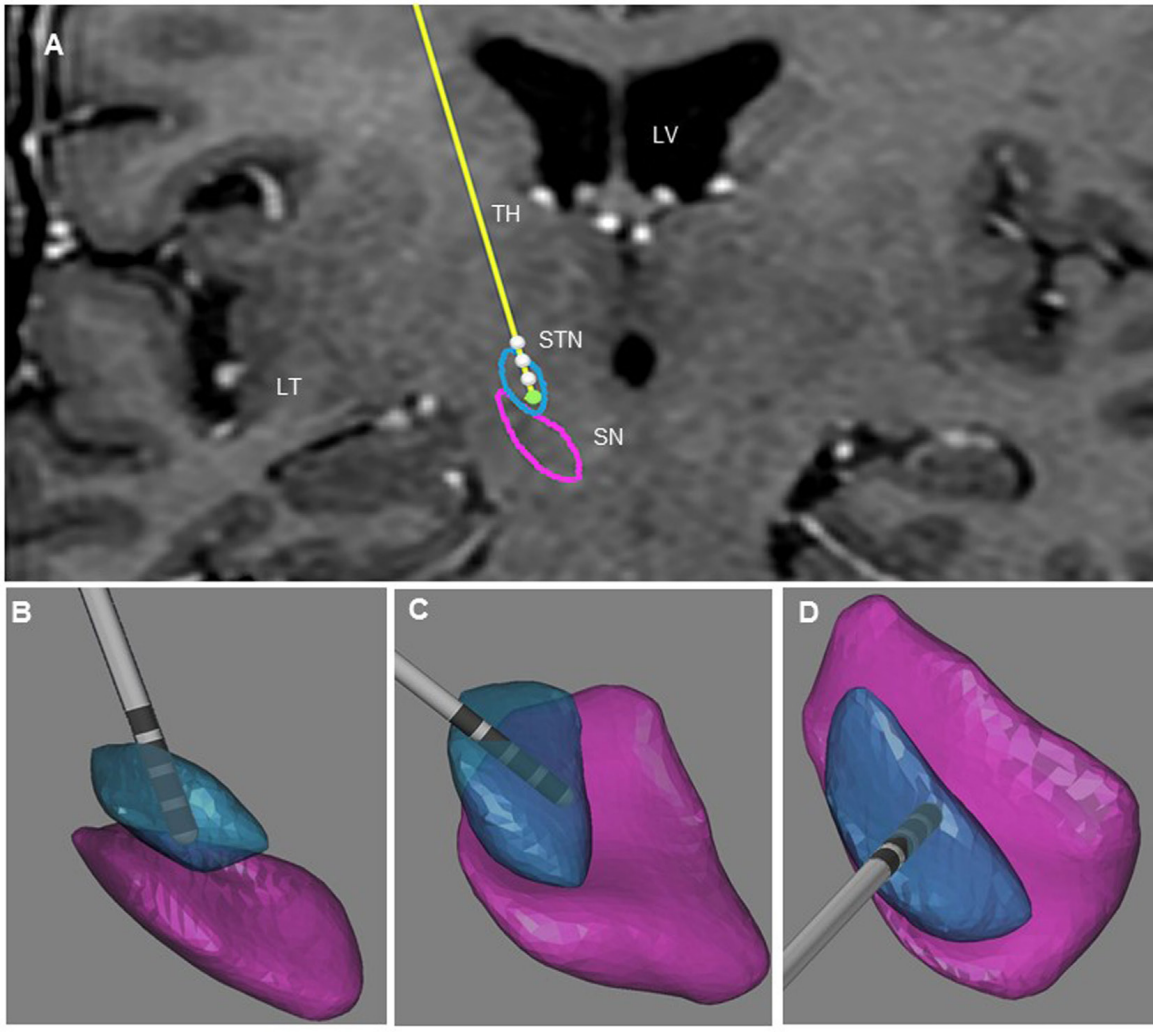
Illustration of the three-dimensional anatomical location of the electrodes (patient 1). **(A)** Frontal view of the electrodes (in yellow) with the different contacts (white) and three-dimensional view of STN (in blue), and SNr (in purple). This representation is superimposed on the coronal T2 preoperative MRI of patient 1 to illustrate the location of the STN and SNr. Each electrode had four contacts with the length of 1.5 mm separated by 0.5 mm intervals and numbered from 0 to 3 in the right hemisphere. Contact 0 is the deepest contact (green). **(B)** Frontal view of the right STN and SNr. **(C)** Anterolateral view of the right STN and SNr. **(D)** Superior view of right STN and SNr. In **(B–D)**, the STN and SN were represented in transparency to show the location of the contacts and visualize the “laughter” contact 0 localized within the ventral and medial STN. LV, lateral ventricle; TH, thalamus; TL, temporal lobe.

**Figure 2:**
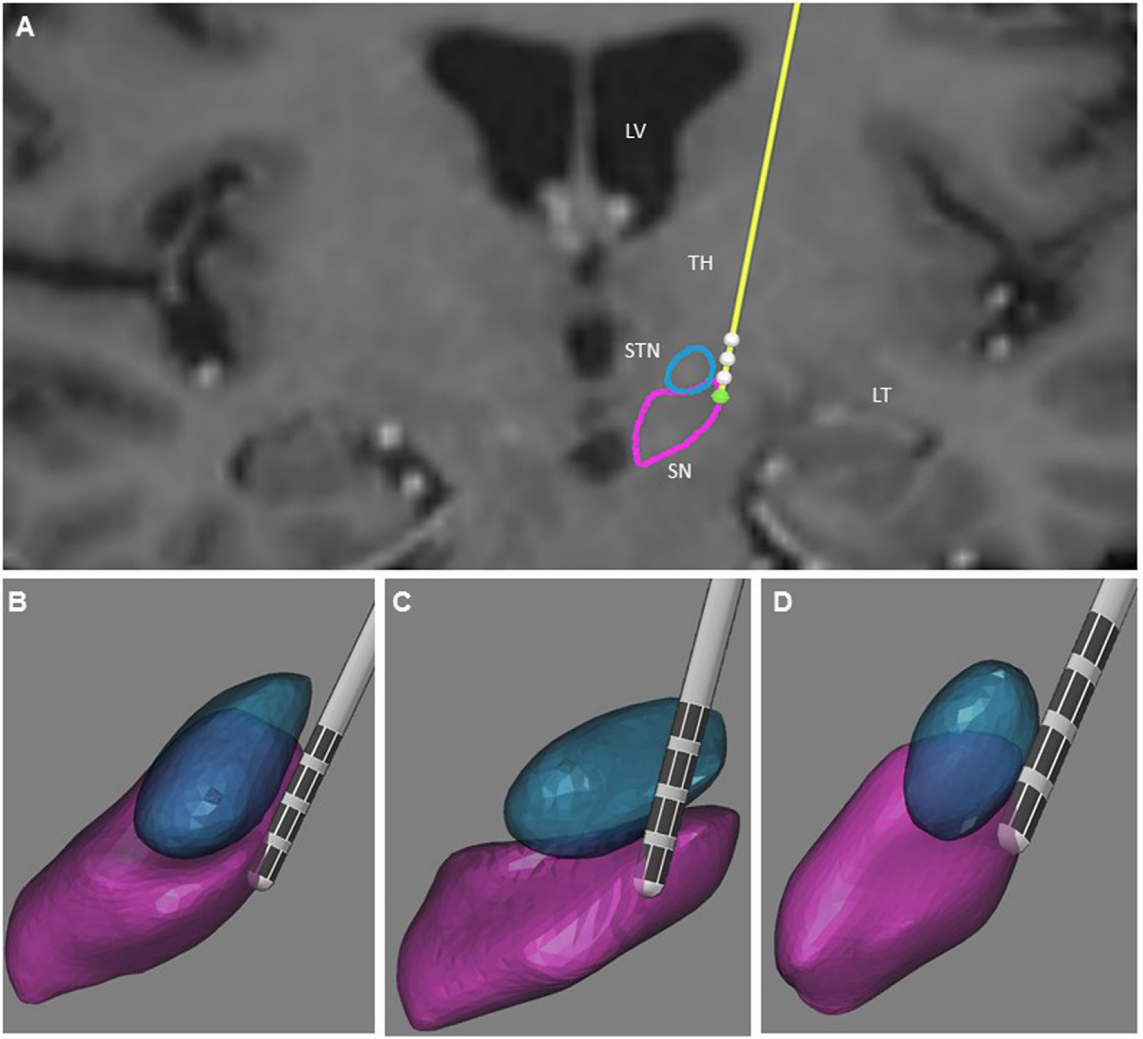
Illustration of the three-dimensional anatomical location of the electrodes (patient 2). **(A)** Frontal view of the electrodes (in yellow) with the different contacts (white) and three-dimensional view of the STN (in blue), and SNr (in purple). This representation is superimposed on the coronal T2 preoperative MRI of patient 2 to illustrate the location of the STN and SNr. Each electrode had four contacts with the length of 1.5 mm separated by 0.5 mm intervals and numbered from 0 to 3 in the left hemisphere. Contact 0 is the deepest contact (green). **(B)** Frontal view of the left STN and SNr. **(C)** Anterolateral view of the left STN and SNr. **(D)** Superior view of the left STN and SNr. In **(B–D)**, the STN and SNr were represented in transparency to show the location of the contacts and visualize the “laughter” contact 0 localized within the SNr. LV, lateral ventricle; TH, thalamus; TL, temporal lobe.

The post-operative outlining and labeling of subthalamic structures were performed through identification of SN and STN between white and gray matters by using stereotactic MRI. The anatomical location of the four contacts was determined after matching post-operative imaging with pre-operative stereotactic MRI (BrainLab, Germany, and WayPoint™ Navigator Software, USA). After matching, the anatomical location was determined contact by contact in a three-dimensional anatomical environment for each patient (Figures 1 and 2). The standard STN coordinates (X, Y, Z) commonly used for indirect targeting are 12 mm lateral from midcommisural point (MCP), 3–4 mm posterior from MCP, and 3–4 mm below intercommissural line. The standard trajectory and approach angles were 60° of anterior angle and 15° of lateral angle from mid-sagittal plane. The trajectory of the electrode in the first patient showed an anterior angle of 59° and lateral angle of 16°. The active contact 0 was located 9.48 mm lateral to MCP, 4.32 mm posterior to MC, and 5.43 mm inferior to MCP. Analysis of the postsurgical images showed that contact 0 was ventrally and medially located electrode within the STN (Figure 1). In the second patient, the trajectory of the electrode demonstrated an anterior angle of 51° and lateral angle of 12°. The anatomical localization of contact 0 was 11.64 mm lateral, but 1.61 mm posterior and 6.10 mm inferior to MCP at the level of the lateral SNr (Figure 2).

## Discussion

Uncontrollable laughter after STN-DBS in PD occurred in two patients with electrodes located more anteriorly, medially, and inferiorly than originally planned. In both instances, uncontrollable laughter appeared acutely with simulation of the most ventral contacts. Post-operative lead location and clinical features suggest that stimulation of the ventral STN or closely related structures accounted for this reproducible emotional side effect. In patient 2, responsible contacts were decisively inferior to the STN (Figures 1 and 2). Uncontrollable laughter has been associated with a direct effect of stimulation of STN or the lesion effect of DBS surgery (9, 11). The STN is a small structure and electrical current can spread from one territory to another especially when high amplitude stimulation is used (6, 12). It is considered an integral part of the indirect pathway by which the striatum controls the output of the basal ganglia and motor function (13, 14). There is evidence indicating that the STN is a critical component of networks controlling motor function as well as cognition and emotion (8, 15, 16). The STN has several functional regions including the dorsolateral area for motor control and the ventral–medial region interfacing with limbic circuits (3, 17). The medial STN is also adjacent to the lateral hypothalamus. Therefore, stimulation of ventral- and medial-STN can impact emotion and cognition networks inducing mood and behavior changes (7, 15, 18). Medial forebrain bundle (MFB) is a key structure of the mesolimbic–dopamine system that is related to affective disorders (19). It is located in the lateral wall of the hypothalamus and bidirectionally connected with the hypothalamus, ventral striatum, accumbens nucleus, and septal area (20, 21). Stimulation of MFB, which is located medially and anteriorly in relation to STN-limbic territories, has been associated with acute hypomania (22, 23). Consequently, stimulation of ventral- and medial-STN can result in spreading of electrical current to co-activate the MBF. The trajectory of the DBS electrodes could affect the anatomical position of the contact. The double oblique trajectory of the quadripolar electrode places the lower contacts more medial and ventral, closer to the limbic STN (3, 6, 24).

Stimulation of limbic-related brain structures outside of STN could also explain the emotional and behavior effects (25). The substantia nigra reticulata (SN) is one of the major dopamine-producing areas of the brain and the main output of the basal ganglia with connections and functions that extend beyond motor control (26, 27). It is also thought to play important roles in behaviors including learning, drug addiction, and emotion. The pars reticulata of the substantia nigra reticulata (SNr) is located at lateral SN (28). It is an important processing center in the basal ganglia. The GABAergic neurons in the SNr convey the final processed signals of the basal ganglia to the thalamus and superior colliculus (29, 30). The GABAergic neurons also spontaneously fire action potentials. In rats, the frequency of action potentials is roughly 25 Hz (31). The purpose of these spontaneous action potentials is to inhibit targets of the basal ganglia, and decreases in inhibition are associated with movement (32). The STN gives excitatory input to the SNr and modulates the rate of firing of these spontaneous action potentials (33). Acute mood change has been reported with stimulation of the SN (26). In this report, the acute mood change in patient 2 was noticed with the stimulation of the most ventral contact in an anteriorly and inferiorly located lead. The active contact anatomical localization is within the SN and probably in the SNr.

Three reports were published regarding uncontrollable laughter after STN-DBS (Table 2). Most case studies report the phenomenon occurring with bilateral STN stimulation (4, 5, 7). All of the reports implicated the most ventral contact as the cause except for one by Krack et al., who reported onset of mirthful laughter in a patient with stimulation of contact 3 from 3.2 V/60 μs/130 Hz to 5.0 V/60 μs/130 Hz on the right side (4). However, no detailed anatomical location of post-operative lead has been reported in previously published cases. When comparing different DBS targets, ventral STN stimulation may lead to more mood and cognitive changes as compared with GPi due to possible spreading of stimulation to the limbic region (34). This is also consistent with our findings that unilateral ventral- and medial-STN stimulation induced acute mood change with uncontrollable laughter. Additionally, cerebral infarctions can cause pathological laughing and crying. Previous reports indicate that post-stroke pathological laughing and crying occurs with bilateral, multiple hemispheric lesions, and in the pons, specifically the bilateral paramedian basal and basal–tegmental areas (35). This is consistent with the postulated-anatomical localization of the centers for facial expression residing in the lower brainstem, along with the thalamus, and hypothalamus (36).

**Table 2:** Clinical characteristics and DBS settings in previous case studies.

Cases study	Location	Active contact	DBS stimulation parameter	Time onset	Medication
1. Chattah et al.	Bilateral STN	Contact 0/1	Monopolar	Acute	OFF
			2.0 V/60 μs/130 Hz		
2. Wojeteki et al.	Bilateral STN	Contact 0/1	Monopolar	Acute	ON
			3.5 V/60 μs/130 Hz		
3. Krack et al.	Bilateral STN	Contact 0	Monopolar	Acute	N/A
			3.6 V/90 μs/160 Hz		
4. Krack et al.	Right STN	Contact 3	Monopolar	Acute	N/A
			5.0 V/60 μs/130 Hz		

*Case 4 is a patient with a history of Parkinson’s disease (PD) and left thalamotomoy, underwent right STN-DBS 3 years after left thalamotomy and presented with acute uncontrolled laughter after right STN-DBS*.

*STN, subthalamic nuclei; N/A, not available*.

## Conclusion

Taken together, our findings suggested that acute uncontrollable laughter can be elicited by stimulation of different limbic structures contiguous to the STN. Our report highlights that, in addition to the limbic STN, the MFB and the SNr might play an important role in the induction of acute mood changes. It is unclear why positive valence was noted with stimulation as opposed to depressed mood. In addition, it would be valuable to monitor if the uncontrollable laughter is replicated at long-term follow up. Stimulation of medially and inferiorly placed contacts or inferiorly and anteriorly located electrodes in relation to the STN should be considered if acute behavioral symptoms are elicited with neurostimulation. Clinicians should consider using more dorsal contacts for DBS programming if this unusual side effect is encountered.

## Ethics Statement

The patients gave the signed consent for releasing her health information in the format of this case report. The authors consulted Albany Medical Center IRB Office. Additional ethics review was not required in this case study.

## Author Contributions

YH and AR-Z analyzed the data and wrote the manuscript. JP and LG collected all the data. JA analyzed the data and made the figures, JD and EM edited the manuscript.

## Conflict of Interest Statement

JP reports consulting for Medtronic, St. Jude, and Boston Scientific; receiving grant support from Jazz Pharmaceuticals, Medtronic, Boston Scientific, St. Jude, and the NIH (grant 1R01CA166379); serving as medical advisor for Centauri; and having stock equity in Centauri. No other disclosures were reported. AR-Z reports consulting for Medtronic and Teva neuroscience. The reviewer MC and handling Editor declared their shared affiliation.
